# Cerebral hemangioma in a 5-month-old Chow Chow dog displaying seizures of acute onset

**DOI:** 10.1186/s13028-026-00872-7

**Published:** 2026-06-19

**Authors:** Pernille Lindholm Heidemann, Michelle Lindeholm Pedersen, Andrew David Miller, Clara Allberg, Jørgen Steen Agerholm

**Affiliations:** 1 Department of Veterinary Clinical Sciences, Faculty of Health and Medical Sciences, University of Copenhagen, Dyrlaegevej 16, Frederiksberg C, DK- 1870 København, Denmark; 2 Department of Population Medicine and Diagnostic Sciences, Section of Anatomic Pathology, Cornell University College of Veterinary Medicine, Ithaca, NY USA

**Keywords:** Brain, Cluster seizures, Ependymal cyst, Ependymal diverticulum, Epilepsy, Histopathology, Intracranial, Juvenile, Neoplasia, Tumor

## Abstract

**Background:**

Cerebral hemangioma is a recognized cause of acute onset of epileptic seizures with interictal neurological abnormalities in dogs, although the number of cases reported is limited. Hemangiomas can affect dogs of all age groups, but there is an increasing risk with age, peaking at nine years.

**Case presentation:**

This case report describes a 5-month-old female Chow Chow dog with an acute onset of epileptic seizures and interictal neurological deficits indicative of a right forebrain lesion due to a cerebral hemangioma and associated hemorrhage. Initially, extracranial causes were excluded, and magnetic resonance imaging of the head was performed, revealing a large well-delineated hemorrhagic mixed intra- and extra axial lobular mass in the right frontal lobe, generalized ventriculomegaly, and two intraventricular smaller, well-defined cyst-like structures in the right cerebellopontine angle and fourth ventricle. The dog was euthanized due to the severity of neurological deficits combined with imaging findings, suggesting a poor prognosis. Post-mortem gross examination, histopathology, and immunohistochemistry identified the larger structure as a hemangioma accompanied by extensive hemorrhage. The significant hemorrhage likely explained the acute onset of neurological signs. It is probable that the hemangioma might have remained subclinical, had this hemorrhage not occurred. The smaller cyst-like structures were identified as multilocular ependymal diverticula originating from the fourth ventricle.

**Conclusions:**

This case report underlines the importance of considering cerebral hemangiomas accompanied by acute hemorrhage as a possible differential diagnosis in cases with an acute onset of epileptic cluster seizures with accompanying interictal neurological signs, here found in a juvenile dog.

**Supplementary Information:**

The online version contains supplementary material available at 10.1186/s13028-026-00872-7.

## Background

In dogs, epileptic seizures are the most common neurological presentation [1, 2], with either an underlying reactive, idiopathic, or structural cause. Structural causes have been reported in 45.1% of dogs with epileptic seizures [2], and as treatment and prognosis differ significantly between underlying idiopathic and structural etiologies [3], differentiation is important to initiate correct treatment and inform owners on prognosis. Importantly, an abnormal interictal neurological examination or cluster seizures significantly increases the likelihood of an underlying structural cause being present [4]. Structural causes include inflammatory lesions, trauma, congenital anomalies, degenerative disease, neoplasia, and vascular disorders [5]. Signalment and clinical presentation will aid in identifying the most likely structural differential diagnoses based on onset and progression, with intracranial vascular disorders generally having an acute onset with no or little progression, and intracranial neoplasia having a more insidious onset and progressive neurological signs [6]. Intracranial neoplasms are more frequent in older dogs, but in younger dogs, one study reported intracranial congenital anomalies and idiopathic epilepsy being equally common causes for epileptic seizures. The same study did not identify intracranial neoplasia in any of the included dog younger < 6 months of age [2], and even the most common intracranial neoplasia in dogs, oligodendrogliomas and astrocytomas, are rarely reported in dogs < 12 months of age [7]. Thus, neoplasia is rarely considered a likely differential diagnosis for acute onset of epileptic seizures in young dogs.

Hemangiomas are defined as benign neoplasms originating from the endothelium with vascular structures close to normal blood vessels [8, 9]. While hemangiomas are a common vascular neoplasia in dogs [9], they are mainly reported in soft tissues, skin, and hemopoietic system [10], while central nervous system (CNS) localization is rare [8]. Currently, only a limited number of case reports are available, especially for cerebral hemangiomas [11–16]. While they can affect dogs of all age groups, there is an increasing risk with age, peaking at nine years [10], with reported cases of cerebral hemangiomas available in literature ranging from 13 months – nine years of age [11–16].

Here, we report a 5-month-old Chow Chow dog with an atypical, acute onset of epileptic cluster seizures and an interictal abnormal neurological examination caused by an underlying cerebral hemangioma with a secondary acute hemorrhage. An ependymal diverticulum was concurrently found affecting the fourth ventricle. Findings were confirmed by histopathology. This case reports an unusual cause for epileptic seizures in a juvenile dog with an atypical acute onset. This rare benign neoplasia may be of relevance to consider as a potential differential diagnosis in acute onset of epileptic cluster seizures, even in young dogs.

## Case presentation

A five-month-old intact female Chow Chow dog, imported from Greece at 15 weeks of age, was referred to the neurology clinic at the University Hospital for Companion Animals (University of Copenhagen, Denmark) with a two-days history of altered mentation, ataxia, vomiting and inappropriate urination indoors. In the last 24 h prior to presentation, the dog had five generalized epileptic seizures and had received an intravenous dose of phenobarbital at an emergency facility.

Upon presentation, the dog was non-ambulatory and obtunded, suggesting either a forebrain- or brainstem lesion. An intermittent right-sided head turn was observed. Postural reactions were absent in all four limbs, with withdrawal reflexes remaining intact. Except for an intermittent positional ventral strabismus on the right eye and bilaterally decreased menace response, cranial nerve examination was normal. Neurological signs, including the history of epileptic seizures, were consistent with a right-sided forebrain lesion, with an additional potential cerebellar or brainstem lesion suspected based on the intermittent positional strabismus present on the right eye.

To rule out extracranial causes, paraclinical examinations were performed. Blood analyses included hematology, within normal reference ranges, serum biochemistry with the only abnormalities being age-appropriate elevations in serum phosphate and alkaline phosphatase, and infectious disease screening for *Anaplasma* spp., *Borrelia burgdorferi*, *Dirofilaria immitis*, *Ehrlichia* spp. (SNAP^®^ 4Dx^®^, IDEXX), and *Angiostrongylus vasorum* (Angio Detect™, IDEXX), all negative. Urine tests for cannabis, opiates, amphetamine, cocaine, methadone, tramadol, buprenorphine, benzodiazepines, fentanyl, ketamine, oxycodone (NanoSticka^®^ Multi-Drug Screen Panel 200 − 37 P12) and nicotine (Cotinine rapid test dipstick, AllTest^®^) were all negative. Given the lack of an identified extracranial cause in combination with neurological signs suggesting a lateralized forebrain lesion, a structural cause was suspected. Differential diagnoses included congenital malformation, meningoencephalitis of unknown origin, infectious encephalitis, a vascular event, or, considered less likely, neoplasia.

**Fig. 1:**
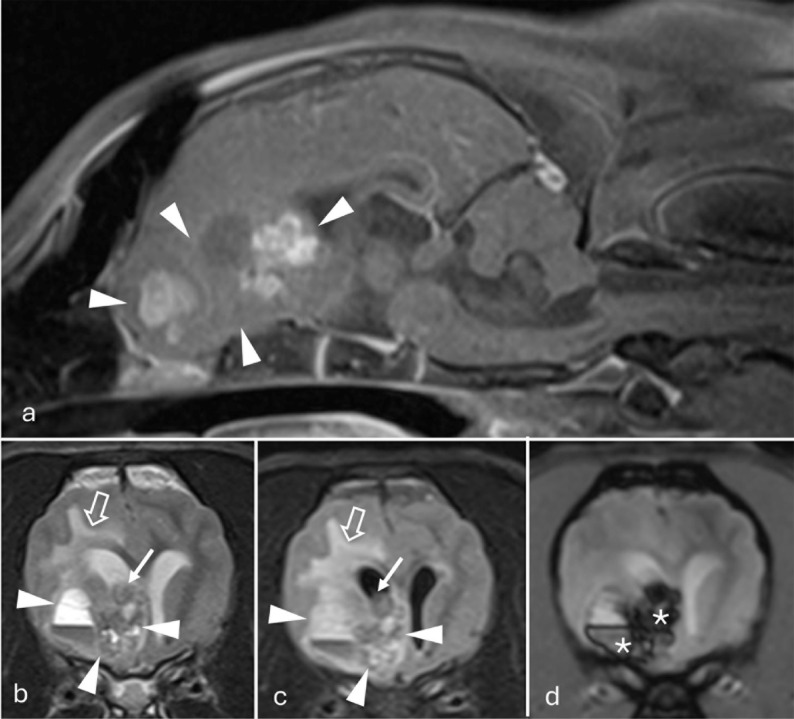
Magnetic resonance imaging findings of the right cerebral mass. **a** Sagittal T1-weighted (T1W) post-contrast image showing an expansile mixed intra- and extra-axial frontal lobe mass with heterogenous signal intensity and heterogenous marked contrast enhancement (arrowheads); **b** Transverse T2-weighted (T2W) image at the level of the caudal aspect of the frontal lobe, and **c** transverse Fluid-attenuated inversion recovery (FLAIR) and **d** transverse T2W Gradient Echo (T2*GRE) at the same level as b) showing a lobulated heterogeneous mass (arrowheads) with extensive haemorrhage (asterix) and suspected intraventricular extension (arrow). A fluid-fluid level separation is noted in the lateral portion of the mass, with T2W, FLAIR and GRE hyperintensity and T1W hypointensity compared to normal grey matter dorsally and T2W and FLAIR hypointensity, T1W isointensity and susceptibility artefact ventrally. The heterogeneous imaging characteristics of the lobulated intra- and extra-axial mass are indicative of varying stages of intralesional haemorrhage. There is extensive perilesional oedema (open arrow)

A high-field magnetic resonance imaging (MRI) system with a head matrix coil (1.5T Siemens Magnetom Altea, Siemens Healthcare A/S, Ballerup, Denmark) was used to obtain images of the neurocranium including T2-weighted (T2W) sagittal and transverse, T1-weighted (T1W) transverse, fluid-attenuated inversion recovery (FLAIR) transverse, T2-weighted gradient echo (T2*/GRE) transverse, diffusion-weighted imaging (DWI) transverse, and T1-weighted post-contrast (T1W + c) multiplanar sequences (Gadovist 1 mmol/mL, 0.1 mL/kg). The MRI revealed a large well-delineated lobulated mass located in the right frontal lobe (Fig. 1), measuring approximately 2.5 by 1.4 cm in size, extending from the olfactory bulb to the rostral aspect of the right lateral ventricle (Fig. 1a). The mass was heterogeneously T1W and T2W hypo- to hyperintense to normal grey matter (Fig. 1a, b), with heterogeneous well-delineated marked post gadolinium contrast enhancement. In GRE sequences the mass demonstrated large regions of heterogeneous susceptibility artefact indicative of extensive intralesional hemorrhage (Fig. 1d). Caudally the mass had invasion into the rostral portion of the right lateral ventricle. A marked mass effect was affecting surrounding structures causing a substantial leftward midline shift. Extensive T2W hyperintensity compared to normal grey matter was tracking along the white matter tracts of the right cerebral hemisphere with evidence of increased diffusion on DWI. Imaging findings of the cerebral mass were compatible with a mixed intra- and extra axial hemorrhagic mass with extensive perilesional vasogenic edema.

**Fig. 2:**
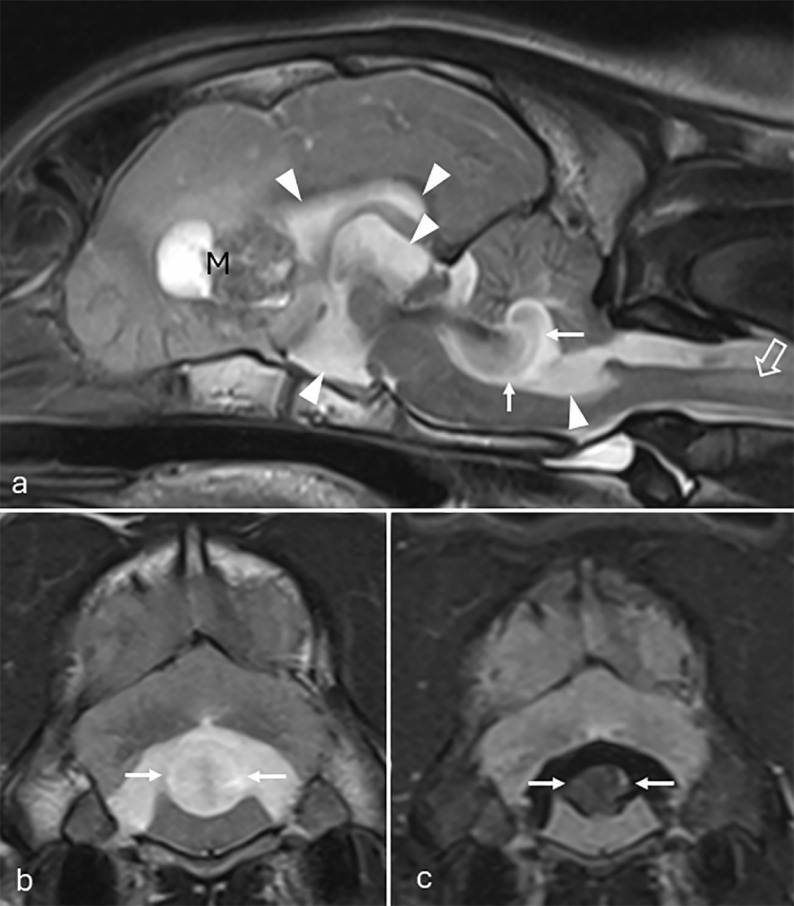
Magnetic resonance imaging findings of ventricular system. **a** Sagittal T2-weighted (T2W), **b** transverse T2W and **c** transverse Fluid-attenuated inversion recovery (FLAIR) image at the level of the fourth ventricle showing a well delineated circular lesion with T2W heterogenous hyperintensity to normal grey matter and partial fluid suppression (arrows). There is generalized ventriculomegaly (arrowheads) and presyrinx formation (open arrow) of the included cervical spinal cord. A lobulated heterogeneously hyper- to hypointense mass (M) compared to normal grey matter is visible in the frontal lobe

The ventricular system was moderately dilated (generalized ventriculomegaly). A poorly delineated fluid signal intense region was affecting the central and dorsal portions of the included cervical spinal cord consistent with presyrinx formation (Fig. 2). An approximately 0.9 cm cyst-like dilation was noted in the right cerebellopontine angle located in the extra axial space, demonstrating a moderate mass effect. The lesion was isointense to cerebrospinal fluid (CSF) and T2W hyperintense to normal grey matter with complete fluid suppression on FLAIR, T1W hypointense to grey matter with no evidence of contrast enhancement or susceptibility artefact. A second well-defined circular lesion (approximately 1.2 cm in diameter) was identified within the central portion of the fourth ventricle. The lesion was T2W hyperintense, T1W hypo- to isointense to grey matter and partially suppressed on FLAIR sequence with no evidence of contrast enhancement or susceptibility artefact.

The dog was euthanized while still under anaesthesia as the neurological status and imaging findings indicated a poor prognosis, and owners did not want to pursue further diagnostics or treatment. The owners gave permission for necropsy. The cadaver was kept at 5 °C until necropsy the following day.

**Fig. 3:**
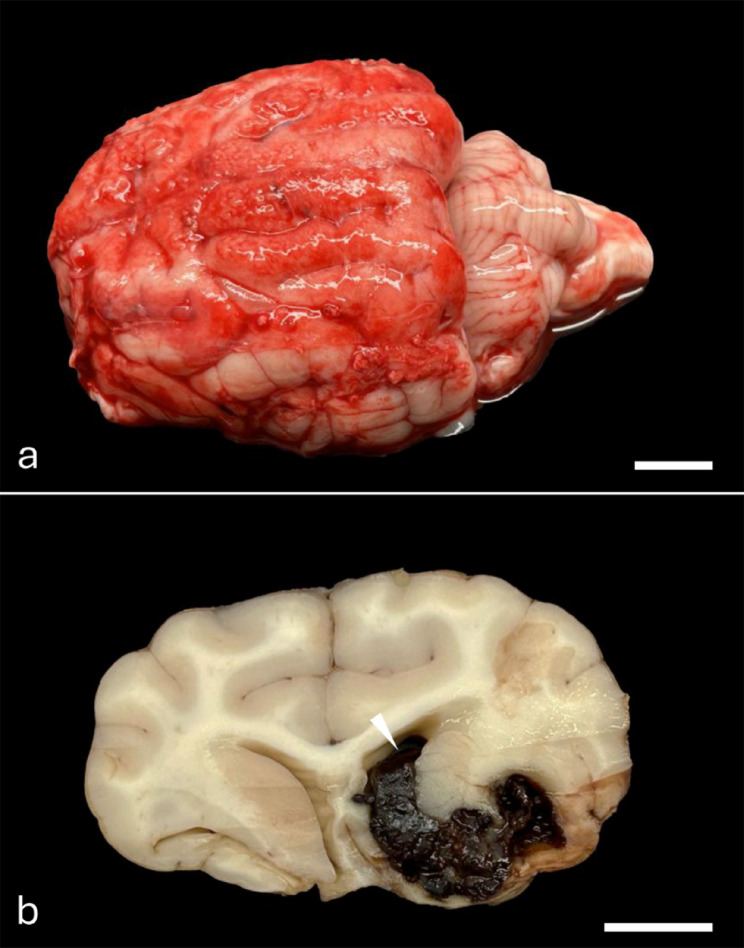
Gross brain lesions. **a** The cerebral hemispheres are covered by erythematous leptomeninges with coalescing, grey plaques. The leptomeninges covering other parts of the brain appeared unremarkable. Bar: 1 cm; **b** An irregularly shaped, expansile, demarcated hemorrhagic mass in the right hemisphere with eruption into the right lateral ventricle. Formalin fixed specimen. Bar: 1 cm.

Necropsy findings were unremarkable except for the brain. Leptomeninges covering the dorsal aspect of the cerebral hemispheres were found thickened with a firm texture. They had a pronounced erythematous appearance, interspersed with coalescing, slightly elevated grey plaques and easily separated from the cerebral cortical surface (Fig. 3a). The leptomeninges covering other parts of the brain and the spinal cord appeared unremarkable.

The entire brain was fixed in 10% neutral buffered formalin for three weeks before further examination as were specimens of lung, heart, liver, spleen, pancreas, kidney, skeletal muscle, and peripheral nerve. The brain was then embedded in agar and cut into coronal 4 mm serial sections. Expanding and compressing a large region of the right cerebral hemisphere was an irregularly shaped, firm, well-demarcated hemorrhagic, multilobular mass. The mass extended into the right lateral ventricle, in which a large, coagulated blood clot was observed (Fig. 3b). The fourth ventricle appeared dilated with slight dorsal displacement of the caudal part of the cerebellum.

All specimens were processed routinely for histology and paraffin embedded. All tissue sections were stained by hematoxylin and eosin while sections of the multilobular mass were additionally stained by Masson’s trichrome, periodic acidic-Schiff, von Kossa and Perl’s Prussian blue stains and immunohistochemically (IHC) stained for glial fibrillary acidic protein (GFAP) (Z0334 Agilent Technologies, Glostrup, Denmark), MAC 387 (anti-MAC 387, clone MCA874G; Bio-Rad, Raleigh, NC, USA), CD31 and smooth muscle actin (SMA) (PA9043, Leica, Buffalo grove, IL, USA). Brain tissue at the level of the fourth ventricle/caudal aspect of cerebellum was stained by Masson’s trichrome and IHC stained for GFAP, cytokeratin (M3515, Agilent Technologies) and vimentin (M0725, clone V9, Agilent Technologies). Details on the IHC methods are available from Additional file 1.

**Fig. 4:**
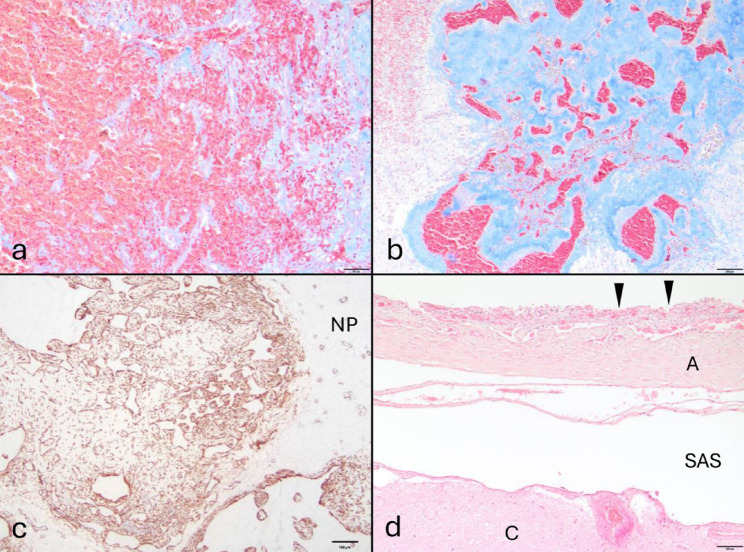
Microscopic characteristics of the cerebral hemangioma. **a** Area of the mass dominated by thin blood vessels and extensive hemorrhage. Masson’s trichrome stain, bar: 50 μm; **b** Area of the hemorrhagic mass dominated by blood vessels with prominent perivascular fibrosis. Masson’s trichrome stain, bar: 100 µm; **c** Periphery of the hemangioma showing variably sized, but mostly small, vascular smooth muscle action labeled channels (capillary-like). Neuroparenchyma (NP; upper right) has an increase in vasculature. Smooth muscle actin immunohistochemistry. Bar: 100 µm; **d** Cerebral cortex (C) covered by a fibrotic arachnoidea mater with vascular proliferation (arrow heads). SAS: Subarachnoid space. HE, bar: 100 µm

The hemorrhagic mass consisted of numerous, variably sized and irregularly shaped, thin-walled blood vessels and extensive hemorrhage. In some areas, groups of blood vessels with prominent perivascular fibrosis were present (Fig. 4a, b). The blood vessels were lined by a single layer of CD31 labeled endothelial cells. Immunohistochemistry for SMA confirmed a thin smooth muscle wall around all blood vessels (Fig. 4c) Numerous hemosiderin-laden MAC 387 labeled macrophages were dispersed in hemorrhagic areas. The neuroparenchyma adjacent to the hemorrhagic mass was necrotic and edematous with a more distant zone of astrocytosis and vascular proliferation. Grossly changed parts of the meninges consisted of a layer of dense collagen filling the subarachnoid space. This layer was covered by numerous thin-walled blood vessels and occasionally irregular calcified bodies (Fig. 4d). The findings were consistent with a cerebral hemangioma with an atypical meningeal spread of proliferative blood vessels.

**Fig. 5:**
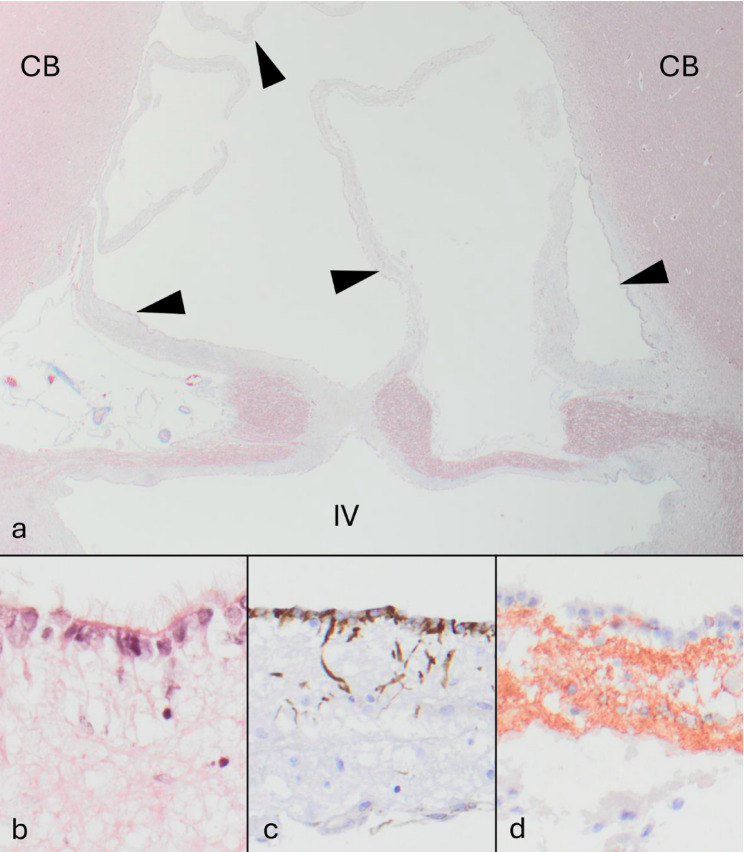
Microscopic characteristics of the ependymal diverticulum. **a** A cystic structure (arrowheads) is located in the cisterna magna region dorsal to the fourth ventricle (IV). The communication to the fourth ventricle is not seen at this level. CB: cerebellum; **b** The inner lining of the systemic structure is composed of a single layer of cuboidal ciliated cells; **c** The epithelial cells of the cystic structure were characterized by marked cytoplasmatic labeling of vimentin. Vimentin immunohistochemistry; **d** The epithelial cells of the cystic structure rested on a dense meshwork of glial fibrillary acidic protein (GFAF) labeled astroglia. A few epithelial cells are labeled with GFAP. GFAP immunohistochemistry

Examination of serial sections of the brainstem revealed a multilocular cystic structure occupying most of cisterna magna (Fig. 5a). The ependymal lining of the fourth ventricle extended continuously through the median aperture and covered the inner surface of the structure. The inner lining consisted of cuboidal epithelial cells having apical cilia (Fig. 5b). The cells labeled for vimentin (Fig. 5c) and occasionally for GFAP but lacked immunolabeling for cytokeratin expression. GFAP immunolabeling revealed that the epithelial cells rested on a dense meshwork of GFAP positive astroglia (Fig. 5d). The epithelial lining of the structure had IHC labeling pattern equal to the ependymal cells of the fourth ventricle thus confirming its ependymal origin. This observed communication with the fourth ventricle led to a diagnosis of an ependymal diverticulum. Additionally, hydromyelia and presyrinx formation in the cervical spinal cord confirmed the imaging findings.

### Discussion and conclusions

Here, we report a 5-month-old Chow Chow dog presenting with acute onset of epileptic cluster seizures and interictal neurological abnormalities. The neurological abnormalities were caused by an unexpected cerebral hemangioma when taking history of onset and signalment into account. However, the acute presentation of neurological signs is less surprising considering the extensive hemorrhage related to the hemangioma. Cases with a similar acute onset due to hemorrhage have been previously reported [12, 13, 15]. The hemangioma might have remained subclinical, at least for some time, had the extensive hemorrhage not occurred. While MRI and post-mortem investigations revealed the presence of generalized ventriculomegaly, ependymal diverticula originating from the fourth ventricle, and hydromyelia and presyrinx in the cervical spinal cord, these findings alone cannot explain the neurological abnormalities and the acute onset. Thus, the extensive hemorrhage, although secondary to the hemangioma, seems to be the primary cause of the acute and early onset in the current case.

The primary consideration of the cerebral mass identified on MRI was a benign congenital or developmental structure with secondary hemorrhage and differential diagnoses such as hemangioma, hamartoma, ruptured aneurysm, or arteriovenous malformation. An inflammatory condition was considered much less likely due to the extensive intralesional hemorrhage. *A. vasorum* had been excluded as a potential cause prior to MRI. Arachnoid or ependymal diverticula were considered likely to explain the cyst-like dilations of the right cerebellopontine angle and fourth ventricle. The clinical significance of the cystic dilations was unclear. The generalized ventriculomegaly and presyrinx formation were attributed either secondary to augmented cerebrospinal fluid flow from the frontal lobe mass, or correlated with the ependymal diverticula, which has been reported previously [17, 18].

The acute onset of epileptic cluster seizures and abnormal interictal neurological signs seen in this dog have also been reported in other canine cases of forebrain hemangioma, with epileptic seizures reported to start 12 h to three days prior to presentation [12, 13, 15]. As in the current case, previous cases found acute hemorrhage related to the hemangioma, likely explaining the sudden onset of neurological signs not classically seen with intracranial tumors [12, 13, 15].

Interestingly, in humans, hemangiomas are one of the most common infantile neoplasms reported with a prevalence of 4.5% [19], and in the CNS, hemangiomas are the third most common primary tumor reported in the age group 14 days – 18 months, accounting for 9% of cases [20]. The underlying pathogenesis seems to relate to dysregulated vasculogenesis and angiogenesis. While the underlying etiology is not completely understood, the dysregulation is suspected to be triggered by hypoxic stress [19]. For ependymal cysts, originating from diverticula as seen in the current case, they can be formed during development of the neural tube, and thus could have been present at birth in the described case [21]. In that scenario, it could be speculated whether the ependymal diverticula caused the generalized ventriculomegaly in the dog reported here, as has been reported in other cases [17, 18]. However, as cysts with ependymal lining can arise secondary to damage like trauma, infarction, or hemorrhage [22], the ependymal diverticula may have developed secondary to the mass effect and generalized ventriculomegaly caused by the cerebral hemangioma.

While the owners elected euthanasia in this case, there are several different treatment approaches that could have been applied for the hemangioma. One case report describes removal of a cavernous cerebral hemangioma in a dog by performing a temporal lobectomy [12], while another reported tumor resection followed by inoculation of an oncolytic virus [15]. In the first case, no tumor recurrence was noted during a 13-month follow-up, while in the second, tumor regrowth was observed, but only after 18 months [12, 15]. Interestingly, in neonatal human cases, treatment of hemangiomas is rarely indicated, as they tend to regress over time, but is recommended if they become life-threatening, for example due to compression of neural structures [19]. In cases where treatment is deemed appropriate, the β-blocker propranolol is suggested as the drug of choice, with a response rate reported in 80–100% of cases [23]. Propranolol is thought to have anti-tumor effects specifically against tumors of vascular endothelial origin, and has been tested in a case series of dogs with hemangiosarcoma (*n* = 3) without any severe adverse reactions [24]. While the dog in the current case was severely affected and obtunded at presentation and thus would have needed treatment with a more immediate effect, propranolol treatment could be an intriguing alternative to surgical resection in less severe clinical cases of hemangiomas within the CNS.

In conclusion, while cerebral hemangiomas have sparsely been reported in dogs previously [11–16], this is the first case in a juvenile dog. Additionally, the dog also had a concurrent ependymal diverticulum. This case describes an acute onset and progression atypical for a tumor, likely due to extensive hemorrhage related to the primary hemangioma, and highlights the importance of considering hemangioma as a potential cause of epileptic cluster seizures, even in very young dogs and with an acute onset of neurological signs.

## Supplementary Information

Below is the link to the electronic supplementary material.

Supplementary Material 1.

## Data Availability

The datasets used and/or analyzed during the current study are available from the corresponding author on reasonable request.

## Abbreviations

CNS: Central nervous system
CSF: Cerebrospinal fluid
DWI: Diffusion-weighted imaging
FLAIR: Fluid attenuated inversion recovery
GFAP: Glial fibrillary acidic protein
GRE: Gradient echo
IHC: Immunohistochemistry
MRI: Magnetic resonance imaging
SMA: Smooth muscle actin
T1W: T1-weighted
T1W + c: T1-weighted post-contrast
T2W: T2-weighted
T2*/GRE: T2-weighted gradient echo
