# Supplementary material for: Cerebral hemangioma in a 5-month-old Chow Chow dog displaying seizures of acute onset
Source: Acta Vet Scand. 2026 Jun 19;68:29. doi: 10.1186/s13028-026-00872-7 (PMC13390147; doi:10.1186/s13028-026-00872-7)
Supplement: Supplementary file 1 — Supplementary Material 1. [file 13028_2026_872_MOESM1_ESM.docx]

**Additional file 1.** Cells markers and immunohistochemical methods used to characterize the cerebral hemangioma and the ependymal diverticulum

| **Cell marker/ manufacturer** | **Clone/host** | **Antigen retrieval/dilution/ chromogen** | **Method** | **Control tissue/species** | **Autostainer** |
| --- | --- | --- | --- | --- | --- |
| Glial fibrillary acidic protein Z0334  Agilent Technologies, Glostrup, Denmark | Polyclonal  Rabbit | Citrate buffer  1:8000  AEC | ImmPRESS^®^HRP goat Anti-Mouse IgG polymer Kit | Brain  Dog | Manual |
| MAC 387  Bio-Rad, Raleigh, NC, USA | Monoclonal MCA874G/MAC387  Mouse | TED-buffer pH 9  1:500  AEC | ImmPRESS^®^HRP goat Anti-Mouse IgG polymer Kit | Lymph node  Pig | Manual |
| CD31  Abcam, Cambridge, UK | Monoclonal SP38  Rabbit | TEG-buffer pH 8  1:100  AEC | Vectastain^®^Elite^®^ABC kit | Tonsil  Pig | Manual |
| Alpha smooth muscle actin PA9043  Leica Biosystems, Buffalo Grove, IL, USA | Monoclonal ASM-1  Mouse | Leica Bond  Ready To Use | Leica Bond  Ready To Use DS9800 | Small intestine  Dog | Leica Autostainer XL, Buffalo Grove, IL, USA |
| Cytokeratin M3515  Agilent Technologies, Glostrup, Denmark | Monoclonal AE1/AE3  Mouse | Protease XXIV  1:2400  DAB | ImmPRESS^®^HRP goat Anti-Mouse IgG polymer Kit | Lung  Dog | Manual |
| Vimentin M0725  Agilent Technologies, Glostrup, Denmark | MonoclonalV9  Mouse | Citrate buffer  1:8000  DAB | ImmPRESS^®^HRP goat Anti-Mouse IgG polymer Kit | Lung  Dog | Manual |
